# Genome-Wide Identification, Expression and Response to Estrogen of Vitellogenin Gene Family in Sichuan Bream (*Sinibrama taeniatus*)

**DOI:** 10.3390/ijms25126739

**Published:** 2024-06-19

**Authors:** Zhe Zhao, Li Peng, Qiang Zhao, Zhijian Wang

**Affiliations:** 1 Integrative Science Center of Germplasm Creation in Western China (CHONGQING) Science City, Southwest University, Chongqing 401329, China; zhaozhe023979@163.com (Z.Z.);; 2 Key Laboratory of Freshwater Fish Reproduction and Development (Ministry of Education), School of Life Sciences, Southwest University, Chongqing 400715, China

**Keywords:** *Sinibrama taeniatus*, vitellogenin, gene family, 17β-estradiol, estrogen receptor

## Abstract

To enhance our understanding of teleost reproductive physiology, we identified six Sichuan bream (*Sinibrama taeniatus*) vitellogenin genes (*vtg1*-*6*) and characterized their sequence structures. We categorized them into type Ⅰ (*vtg1*,*4*,*5* and *6*), type Ⅱ (*vtg2*) and type Ⅲ (*vtg3*) based on differences in their subdomain structure. The promoter sequence of *vtgs* has multiple estrogen response elements, and their abundance appears to correlate with the responsiveness of *vtg* gene expression to estrogen. Gene expression analyses revealed that the vitellogenesis of Sichuan bream involves both heterosynthesis and autosynthesis pathways, with the dominant pathway originating from the liver. The drug treatment experiments revealed that 17β-estradiol (E_2_) tightly regulated the level of *vtg* mRNA in the liver. Feeding fish with a diet containing 100 μg/g E_2_ for three weeks significantly induced *vtg* gene expression and ovarian development, leading to an earlier onset of vitellogenesis. Additionally, it was observed that the initiation of *vtg* transcription required E_2_ binding to its receptor, a process primarily mediated by estrogen receptor alpha in Sichuan bream. The findings of this study provide novel insights into the molecular information of the vitellogenin gene family in teleosts, thereby contributing to the regulation of gonadal development in farmed fish.

## 1. Introduction

Vitellogenin (Vtg) is a large phospholipoglycoprotein that serves as a precursor to yolk protein (YP) and is essential for reproduction in oviparous animals [1]. The source of Vtg can be categorized into autosynthesis and heterosynthesis pathways based on its site of synthesis [2]. It is generally accepted that heterosynthetic synthesis in the liver represents the primary mode of vitellogenesis in teleosts, whereby Vtg undergoes translation followed by glycosylation, phosphorylation and lipid modification and ultimately is released into the bloodstream as homomeric complexes [3]. The Vtg is then transported to the ovary through the circulatory system, where it enters the developing oocytes through receptor-mediated endocytosis and undergoes proteolytic cleavage by cathepsins into multiple small YPs with specialized functions [4]. Numerous studies have demonstrated that the five YP domains of teleosts include lipovitellin heavy chain (LvH), phosvitin (Pv), lipovitellin light chain (LvL), β′-component (β′-c) and C-terminal peptide (Ct) [1,5].

Vtg is encoded by a variable number of paralog gene families, and the diversity of Vtgs has been extensively confirmed and explored in past studies [6,7,8]. The current understanding suggests that teleosts produce multiple variants of Vtg, as evidenced by the discovery of 8 Vtg genes in zebrafish (*Danio rerio*) [9] and up to 20 Vtg genes in rainbow trout (*Oncorhynchus mykiss*) [10]. The evolutionary history of the vitellogenin gene family has been extensively studied, and it is widely believed that the presence of multiple copies of vitellogenin in the genome can be attributed to whole genome duplication (WGD) events [11]. Vertebrates underwent four rounds (R) of WGD, including 1R and 2R that existed before the evolutionary differentiation of vertebrates, 3R that occurred in teleosts and subsequent 4R experienced specifically by salmonids. Theoretically, teleosts should possess a total of eight Vtg genes. However, the quantity of Vtg may vary among species due to events such as lineage-specific gene losses and duplications, which could concurrently confer new functions on Vtg [12,13]. In addition to serving as a nutrient reserve, vitellogenin-derived YPs have been shown to regulate egg buoyancy by participating in oocyte hydration [14,15]. In addition, Vtgs have been found to play a pivotal role in physiological processes, such as antimicrobial [16], immune [17] and antioxidant activities [18] in fish. These processes appear species-specific, so studying Vtgs’ structures in different species can enhance our comprehension of ovarian development, regulatory processes, structure-function correlations and environmental adaptation.

In oviparous vertebrates, the process of synthesizing and accumulating yolk nutrients in the body is known as vitellogenesis [19,20]. The vitellogenesis process is dynamically regulated by the hypothalamic–pituitary–gonadal (HPG) axis, producing estrogen that enters hepatocytes and then initiates the transcription of the Vtg gene [21,22]. The response of vitellogenesis to estrogen and its analogues is well recognized [23,24,25]. However, the fish Vtg family comprises numerous members with variations in structural domains, and more comprehensive research is needed on the effects of estrogen on different types of Vtgs. Estrogen requires binding to its receptors to exert its biological effect, and now, at least three nuclear estrogen receptors (nERs) have been identified in teleosts, including Erα, Erβ1 and Erβ2 [26,27]. The roles of nERs in vitellogenesis have been controversial despite extensive in vivo and in vitro studies across various model systems. Studies conducted in various species, including mice [28], *Micropterus salmoide* [29], *Oreochromis mossambicu* [30] and *Pimephales promela* [31], have demonstrated that estrogen induces Vtg transcription primarily by binding to Erα. However, a report on rainbow trout showed that the treatment of primary hepatopancreatic cells with Erβ agonists induced vitellogenesis in contrast to ERa agonists, which did not produce the same effect [32]. This finding highlights the significant role of Erβ as a central mediator in the production of vitellogenin. Moreover, a study using specific silencing RNAs (siRNAs) to target the ER subtypes of goldfish revealed the essential roles of both Erα and Erβs in vitellogenesis and observed that ERβs enhanced Vtg gene transcription by upregulating the expression of ERα [26]. These findings suggest a potential synergistic impact of ER subtypes on fish vitellogenesis, although this hypothesis has yet to be fully substantiated.

Sichuan bream (*Sinibrama taeniatus*) is a small economic Cyprinidae fish endemic to the upper reaches of the Yangtze River. In our recent study [33], we have identified the boundaries of the various stages of Sichuan bream oogenesis and further investigated the key factors involved in its vitellogenesis. However, there still needs to be more molecular information on the Vtg family of Sichuan bream and their specific contribution to vitellogenesis. In this study, we conducted a comprehensive genome-wide investigation of the vitellogenin gene family in Sichuan bream, systematically examined their molecular and structural characteristics and compared them with the peptide sequences of other oviparous species. Additionally, we assessed the expression profiles of *vtgs* across various tissues and stages of ovarian maturation. Finally, we analyzed the specific responses of *vtgs* to estrogen and nERs by incorporating drugs into the diet. The present study offers a comprehensive insight into the molecular information of vitellogenin in Sichuan bream, elucidating their potential roles in the process of vitellogenesis and providing a valuable reference for regulating ovarian development in economically important fish species.

## 2. Results

### 2.1. Identification, Localization and Promoter Sequence Analysis of Sichaun Bream Vtgs

**Figure 1:**
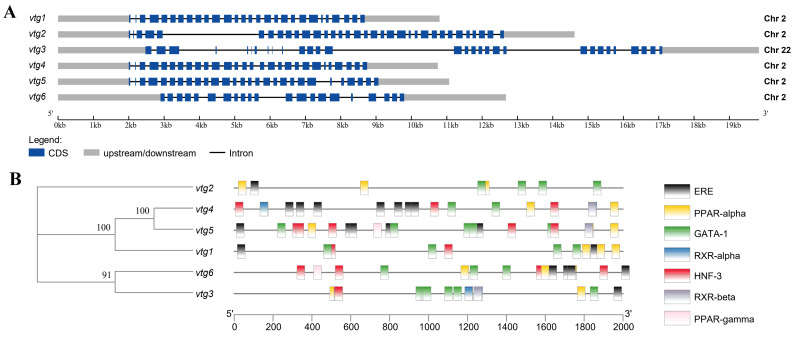
The gene structure of Sichuan bream vitellogenin. (**A**) A schematic representation of the genomic structure of *vtg1*–*6* (drawn to scale); (**B**) Promoter sequence analysis diagram. The phylogenetic tree based on *vtg* promoter sequences is shown on the left, and the corresponding distribution of predicted cis-acting elements is shown on the right.

A total of six vitellogenin genes were identified through a comprehensive search of *Sinibrama taeniatus* genome database (unpublished). We named them *vtg1*–*6* and submitted their corresponding cDNA sequences to NCBI GenBank (*vtg1*, OR759787; *vtg2*, OR759788; *vtg3*: OR759789; *vtg4*: OR759790; *vtg5*: OR759791; *vtg6*: OR759792). The Sichuan bream Vtg CDS sequence length ranged from 3342 to 4848 bp, and the number of amino acid residues of the deduced peptide ranged from 1113 to 1615 aa, corresponding to isoelectric points (pI) and molecular weights (Mw) ranging from 7.00 to 9.12 and 121.99 to 177.07 kDa (Table 1). The gene structure analysis revealed that *vtg1*, *2*, *4*, *5* and *6* were all clustered on chromosome 2. At the same time, only *vtg3* was located on chromosome 22, all of which were interrupted by multiple introns (Figure 1A). We analyzed the promoter region for Sichuan bream *vtg* genes and employed stringent criteria to acquire transcription factor information (Figure 1B). The results revealed a high homology in the promoter sequences between *vtg1*, *vtg4* and *vtg5* as well as between *vtg3* and *vtg6*. All *vtgs* have potential copies of the imperfect estrogen response element (ERE) in their promoter regions, with *vtg3* and *vtg2* having only one and vtg4 containing up to seven. Based on previous research findings [31,34,35,36], we also identified several potential cis-acting elements, such as PPARs, GATA-1, RXRs and HNF-3, which may have special significance in regulating Vtg transcription.

### 2.2. Amino Acid Sequence Analysis

In order to further determine the identification, classification and characterization of Sichuan bream Vtgs, the deduced peptides were subjected to amino acid sequence analysis. The N-terminal region of Vtg1,2,4 and 5 were predicted to have a signal peptide composed of 15 amino acids. Multiple comparisons with zebrafish (*Danio rerio*) Vtgs (Supplementary Figure S1) revealed that Vtg1, 4, 5 and 6 belong to the VtgAo1 type (type Ⅰ) and contain the LvH, Pv and LvL domains. Vtg2 belongs to the typical VtgAo2 type (type Ⅱ), containing five complete YP domains. Vtg3, lacking Pv, β′-c and Ct domains, belongs to the VtgC type (type Ⅲ) and consists only of LvH and LvL. It is noteworthy that Vtg6, which contains the most minor amino acid residue and molecular weight, is classified as type Ⅰ due to its possession of LvH, Pv and LvL domains, even though they are comparatively diminutive in size compared to other type I Vtgs.

The complete Vtg2 has several known conserved motifs, including the vertebrate conserved RGILN motif in LvH and the CGLC motif present in the β′-c domain. Conserved peptide cleavage sites between LvH and Pv (KLKKIL) and between β′-c and Ct (QEY) were also found in Vtg2, but the second and third lysines in KLKKIL were replaced by arginine and glutamate, respectively (KLREIL). In addition, 14 conserved cysteine residues were found in the β′-c and Ct domains of Vtg2. In type Ⅰ Vtgs, RGILN motifs located in LvH and KLREIL motifs between LvH and Pv were detected (lacking RGILN in Vtg6). Moreover, type Ⅲ Vtg only has RGILN motifs.

In addition, multiple phosphorylation sites were detected in each of the peptide sequences in this study, with Vtg6 containing a minimum of 95 and Vtg2 containing a maximum of 127. The number of O-glycosylation sites exhibited significant variation across the polypeptide sequences, with type Ⅲ Vtg3 displaying the lowest count of 13 and type Ⅰ Vtg1 exhibiting the highest count of 37. Additionally, only the 1 N-glycosylation site was detected in Vtg3. It is worth noting that except for Vtg3 lacking a Pv structural domain and Vtg6 having a relatively low molecular weight, the remaining Vtgs exhibit a significant proportion of serine residues, ranging from 44% to 50%, most of which are predicted as potential phosphorylation and O-glycosylation sites.

### 2.3. Homology and Phylogenetic Analyses

Several representative fish Vtg sequences were selected for a sequence consistency comparison analysis with Vtg sequences of various types of Sichuan bream, and the results showed a high level of homology among Sichuan bream type Ⅰ Vtgs (>97%), except for Vtg6. The homology between the type Ⅰ Vtgs and Vtg2 was 72.9–73.2%, and that with Vtg3 was 23.1–23.4%. In addition, type Ⅰ Vtgs showed high homology with carp (*Cyprinus carpio*) VtgAo1 and zebrafish Vtg1, both exceeding 82.4% and between 41.5% and 44.3% with VtgAa in croaker (*Larimichthys crocea*) and bass (*Morone saxatilis*). The homology of type Ⅱ Vtg with carp VtgAo2, zebrafish Vtg2, croake VtgAb and bass VtgAb was 87.8%, 80.5%, 51.9% and 52.6%, respectively. Moreover, the sequence homology of type Ⅲ Vtg with carp VtgC, zebrafish Vtg3, croaker and bass VtgC were observed to be at levels of 80.1%, 76.4%, 45.1% and 45.6%, respectively (Supplementary Table S1).

**Figure 2:**
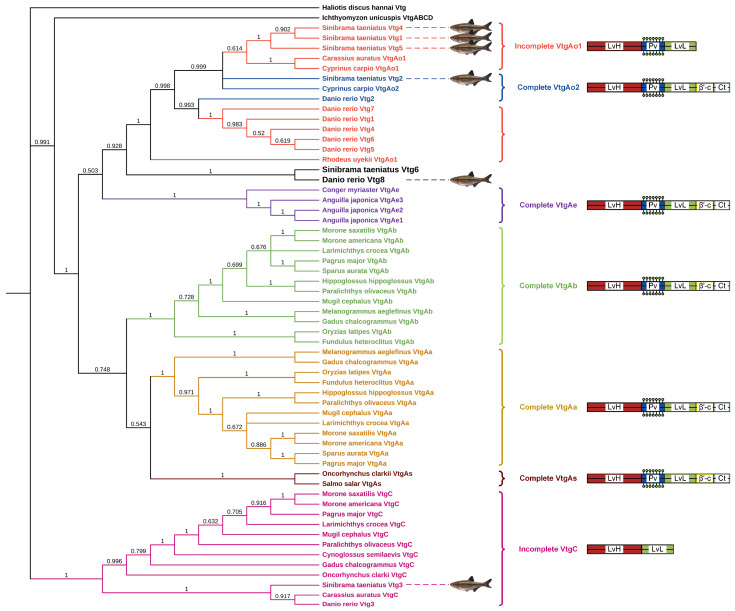
Phylogenetic tree based on Vtg polypeptide sequences from Sichuan bream and several other oviparous vertebrates. The right panel displays the YP domains of each Vtg type (not drawn to scale).

Furthermore, the Vtg amino acid sequence of the oviparous invertebrate *Haliotis discus hannai* was employed as the outgroup to construct a neighbor-joining phylogenetic tree of Vtg peptides from Sichuan bream and various other oviparous vertebrates (Figure 2). Based on differences in YP domains, the three types of Vtgs in Sichuan bream were divided into three clusters, with type Ⅰ Vtgs and Vtg2 having a closer evolutionary relationship with each other than Vtg3. We noticed that the topology of the phylogenetic tree is consistent with the classical taxonomic structure and that Sichuan bream is mainly classified as a clade with carp, zebrafish and *Rhodeus uyekii*, which belong to the family Cyprinidae of the order Ostariophysi. Interestingly, the Sichuan bream Vtg6 and zebrafish Vtg8, belonging to type Ⅰ and type II Vtg, respectively, were grouped in a single clade, which could be attributed to their possession of shorter YP domains compared to other members of the same type. The VtgAo of Ostariophysi clustered into a single clade and then clustered with the VtgAe of Elopomorpha into a large clade. At the same time, VtgAa and VtgAb of Actinopterygii and VtgAs of Salmoniformes were also clustered into a large clade. The two large clades were clustered together with the VtgABCD of *Ichthyomyzon unicuspis*, and finally, VtgC was clustered in the cluster.

### 2.4. Tissue Distribution of vtg Transcripts

**Figure 3:**
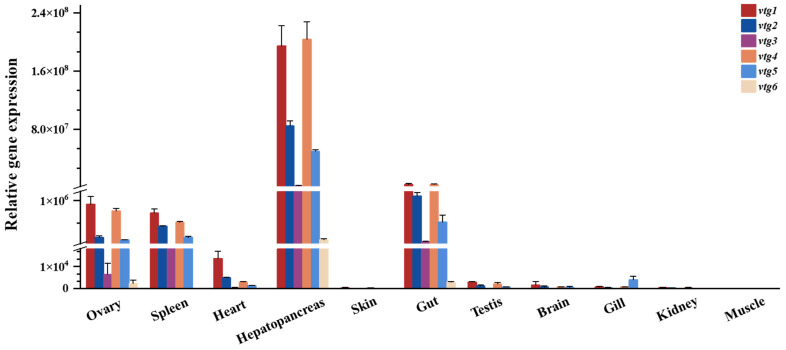
The relative expression of Sichuan bream vitellogenin genes in various tissues.

The transcript levels of *vtgs* among different tissues of adult Sichuan bream were analyzed with qRT-PCR (Figure 3), and the results revealed that the expression of *vtgs* was obviously tissue specific. The liver (hepatopancreas) exhibits the highest gene expression level, followed by the gut, where the relative expression of *vtgs* is 24-94 times greater than in the gut. A significant expression of *vtgs* was also detected in the ovary, spleen and heart, with minimal relative expression of *vtgs* in the other tissues tested. Among all the tissues showing apparent *vtg* expression, type Ⅰ *vtgs* predominated, particularly *vtg1* and *vtg4*, which contributed nearly equal amounts of substantial expression except in the heart. Subsequently, *vtg2* and *vtg3* followed in that order. It is noteworthy that vtg6, characterized by the lowest molecular weight, exhibits the most minor expression level across all tissues.

### 2.5. Expression of vtgs in Liver and Ovary at Different Developmental Stages

**Figure 4:**
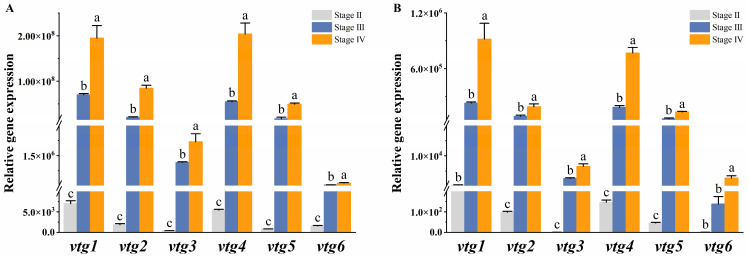
The relative expression of Sichuan bream vitellogenin genes at different developmental stages. The relative expression of Sichuan bream vitellogenin genes in the (**A**) liver and (**B**) ovary from ovarian development stage Ⅱ to stage Ⅳ. ^a, b, c^ Mean values with unlike letters were significantly different (*p* < 0.05).

We further investigated the expression profiles of *vtgs* in the liver and ovary at different developmental stages to elucidate their contribution to vitellogenesis. The results demonstrated a similar expression pattern of *vtgs* between the two tissues, exhibiting a weak expression prior to vitellogenesis (stage II) and a rapid surge during the active phase of vitellogenesis (stage III), ultimately peaking in late vitellogenesis (stage IV). The liver underwent dramatic changes during the vitellogenesis stage, with a consistent and significant increase in the expression of all *vtgs* observed at both stage III and stage IV (*p* ˂ 0.05) (Figure 4A). In the ovary, the expression of *vtg2*, *vtg3*, *vtg4* and *vtg5* increased significantly with different developmental stages (*p* ˂ 0.05), while the expression of *vtg1* and *vtg6* was significantly upregulated only at stage IV (*p* ˂ 0.05) (Figure 4B).

### 2.6. Effects of Estrogen and Selective Estrogen Receptor Modulators on Ovarian Development and Vtg Expression

**Figure 5:**
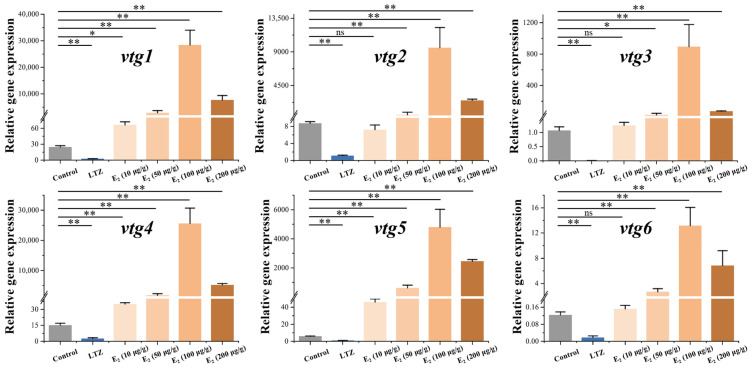
Effect of letrozole (LTZ) and different concentrations of E_2_ treatments on the relative expression of Sichuan bream vitellogenin genes in the liver. * *p* < 0.05; ** *p* < 0.01; ^ns^ means values between the comparison groups were no significantly different (*p* > 0.05).

In order to investigate the regulatory effect of estrogen on vitellogenesis, we conducted drug treatment experiments on juvenile Sichuan bream that developed up to 100 days. After three weeks of feeding with chemical-added feed, we examined the relative expression of *vtg* mRNA in the liver (Figure 5). The results demonstrated the significant suppression of the expression of all *vtgs* following treatment with the E_2_ antagonist letrozole (*p* ˂ 0.05). In addition, we found a significant dose effect of E_2_ on the stimulation of *vtg* expression at dose intervals of 10, 50 and 100 μg/g. However, *vtg* expression was significantly decreased at E_2_ doses up to 200 μg/g. When E_2_ was added to the feed at a concentration of 10 μg/g, only the expression of *vtg1*, *vtg4* and *vtg5* demonstrated a significant upregulation compared to the control group (*p* ˂ 0.05). At the same time, there were no significant changes in the expression levels of other *vtgs*. All vtgs showed significant upregulation when higher concentrations of E_2_ were incorporated into the diet (*p* ˂ 0.05). The most pronounced promoting effect was observed in the 100 μg/g group, where the expression of *vtgs* in the liver significantly increased from 106 to 1683 times compared to the control group.

**Figure 6:**
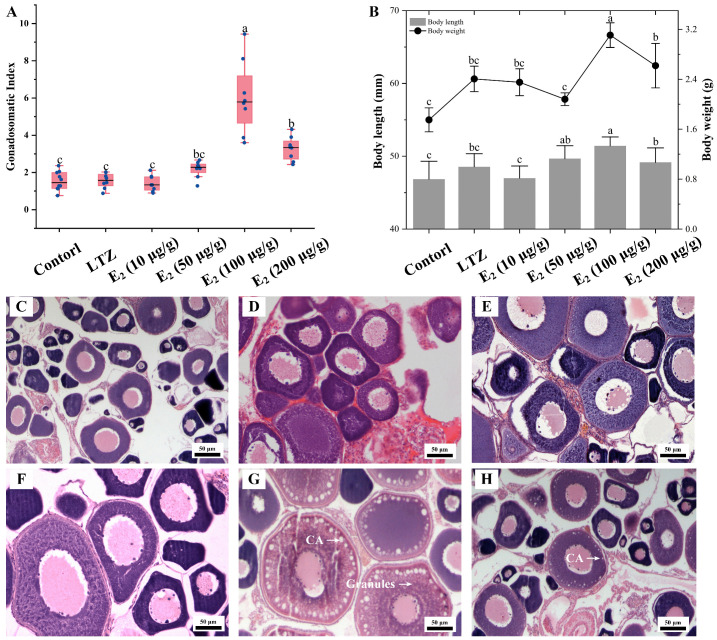
Effect of letrozole (LTZ) and different concentrations of E_2_ treatments on the ovarian development of Sichuan bream. (**A**) Gonadosomatic index (GSI) among different groups. (**B**) Growth parameters among different groups. ^a, b, c^ Mean values with unlike letters were significantly different (*p* < 0.05). Ovarian tissue sections corresponding to the (**C**) control group and under (**D**) letrozole, (**E**) E_2_ (10 μg/g), (**F**) E_2_ (50 μg/g), (**G**) E_2_ (100 μg/g) and (**H**) E_2_ (200 μg/g) treatments, respectively. Arrows mark cortical alveoli (CA) or yolk granules.

The impact of estrogen on vitellogenesis was further investigated through growth parameter measurements and histological observations. Consistent with the qRT-PCR results, the most significant effect was observed after three weeks of feeding with E_2_ supplemented at 100 μg/g food, and Sichuan bream ovaries exhibited the highest GSI. Among the other groups, the GSI in the E_2_ (200 μg/g) group was significantly smaller than that in the E_2_ (50 μg/g) group but significantly larger than that in the control, LTZ and E_2_ (10 μg/g) groups (Figure 6A). The distribution trends of body length and body weight were similar, with the E_2_ (100 μg/g) group being significantly larger than the other groups (*p* ˂ 0.05), followed by the E_2_ (200 μg/g) group. There was no significant difference between the control, LTZ and E_2_ (10 μg/g) groups (Figure 6B). Histologically, it was observed that oocytes in the E_2_ (100 μg/g) group were full of rich yolk vesicles (or cortical alveoli) and granules, indicating that the ovaries had developed to the stage of vitellogenesis (Figure 6G). Only one layer of cortical vesicles was observed at the edge of the oocytes in the E_2_ (200 μg/g) group, indicating that ovarian development was at an early stage of vitellogenesis (Figure 6H). In addition, no significant changes were observed in the histologic characteristics of the ovaries in the other groups that were still in the pre-vitellogenic stage (Figure 6C–F). 

**Figure 7:**
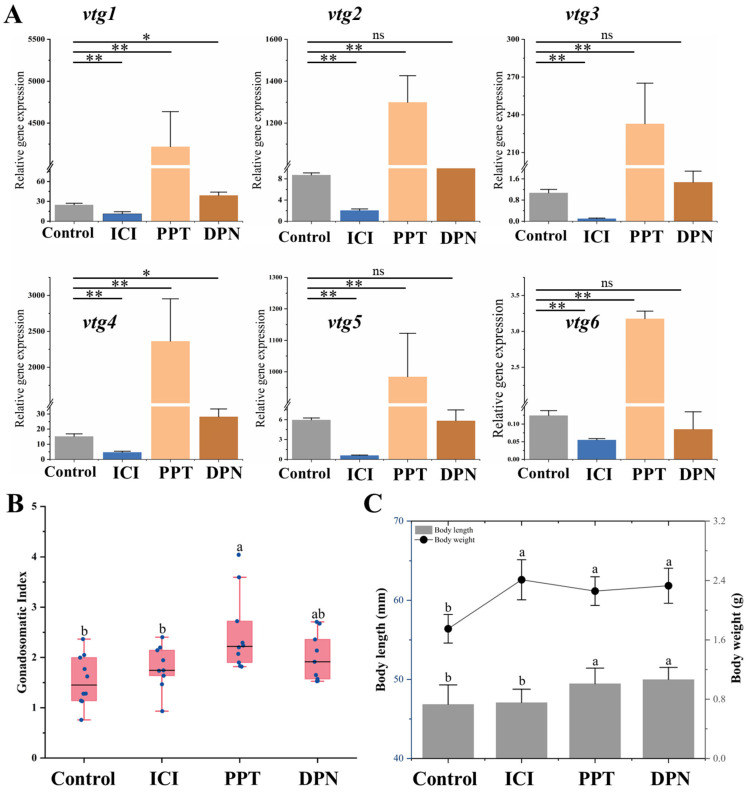
Effects of SERMs on vitellogenesis of Sichuan bream. (**A**) The relative expression of the vitellogenin genes in the liver of Sichuan bream after ICI, PPT and DPN treatments; * *p* < 0.05; ** *p* < 0.01. (**B**) Gonadosomatic index (GSI) among different groups. (**C**) Growth parameters among different groups. ^a, b^ Mean values with unlike letters were significantly different (*p* < 0.05).

In order to elucidate the specific role of nuclear estrogen receptors (nERs) in regulating vitellogenesis, a 3-week feeding experiment was conducted using a feed supplemented with 100 μg/g selective estrogen receptor modulators (SERMs). The findings demonstrated distinct response patterns of *vtgs* in the liver towards these chemicals. It was observed that after treatment with nERs antagonist ICI, the expression of all Vtgs’ mRNA was significantly inhibited (*p* ˂ 0.05). The compound PPT acts as a specific agonist of ERα, leading to a significant upregulation in the expression levels of all *vtgs* by 26 to 170 times. It is noteworthy that the specific agonist DPN, targeting ERβ, only induces a modest increase of 1.6 and 1.9 times in the expression levels of *vtg1* and *vtg4* (Figure 7A). In addition, the administration of these SERMs did not cause significant changes in ovarian histological characteristics (Supplementary Figure S2). However, the GSI after PPT treatment exhibited a significantly higher value than the control and the ICI-treated groups (*p* ˂ 0.05). There was no significant difference between GSI treated with DPN and other groups (*p* > 0.05) (Figure 7B). In terms of growth parameters, the body length of both estrogen receptor agonist-treated groups was significantly greater than that of the control and inhibited groups (*p* ˂ 0.05). The body weight of the control group was significantly smaller than that of the other groups (*p* ˂ 0.05) (Figure 7C).

## 3. Discussion

### 3.1. Structure and Function of Vtgs

Vitellogenesis is the most critical stage in fish oogenesis and provides the nutritional basis for the survival and development of the offspring [37]. To enhance our understanding of vitellogenesis in the Sichuan bream, we conducted a comprehensive genome-wide identification and characterization of the vitellogenin gene family, assessed their expression levels across various tissues and developmental stages and investigated their response to estrogen and estrogen receptors through drug treatment experiments. The present study identified six *vtg* genes, all of which were located on chromosome 2 except for *vtg3*, which was found on chromosome 22. This pattern of chromosome localization is highly conserved among oviparous vertebrates [3,38]. According to a recent report, the chromosome containing multiple *vtg* genes is referred to as the M region, while the chromosome containing only the type Ⅲ *vtg* gene is referred to as the S region [12], which may derive from lineage-specific duplications occurring in teleost genomes (TSGD) that are associated with further rearrangements [39,40]. The identified Vtgs can be categorized into three types based on sequence structural features. Vtgs from type Ⅱ (Vtg2) to type Ⅰ (Vtg1, 4, 5, 6) to type Ⅲ (Vtg3) correspond to intact Vtg, those lacking β′-c and Ct domains Vtgs and those lacking Pv, β′-c and Ct domains, respectively. According to the authoritative “3R hypothesis” [11], they are homologous to the acanthomorph teleosts VtgAb, VtgAa and VtgC, respectively. The identification and classification we proposed were further supported by the results of phylogenetic analyses, which revealed that Vtgs with structural differences formed distinct clades.

It is worth noting that *vtg6*, although nominally classified as a type Ⅰ Vtg, possesses the LvH domain that is missing a large number of amino acids compared to other type Ⅰ Vtgs, which results in low sequence similarity between *vtg6* and other *vtgs*. Phylogenetic analyses showed that Sichuan bream Vtg6 and zebrafish Vtg8 are phylogenetically close to each other, possibly because they both exhibit miniaturization and incompleteness in the LvH structural domain [9]. The expression levels of Vtg6, similar to zebrafish Vtg8, were found to be the lowest in subsequent expression analyses, suggesting that this small Vtg has a limited contribution to vitellogenesis. Further investigation is needed to validate the evolutionary origin and functionality of the vtg6 gene. The natural stepwise deletions of β′-c, Ct and Pv domains in the Vtgs of Sichuan bream and even the absence of part of the LvH domain in Vtg6 make Sichuan bream a potentially good model for studying the Vtg structure–function relationship in Cyprinidae fish.

The molecular characterization of Vtgs is essential for understanding the molecular basis involved in the structure and function development of the gonads [41,42]. It has been found that lipovitellin (LvH + LvL, Lv) consists of amphipathic structures possessing a basket formed with a lumen of hydrophobic residues to hold and transport lipids [8]. In addition, Lv carries a Vtg receptor-binding peptide (VRBP), which is closely related to the Vtg recognition process that occurs on the oocyte surface [43]. Lv was detected in all Sichuan bream Vtgs, as it represents the fundamental structure of Vtg and often constitutes the majority of the Vtg mass. We found that the proportion of serine residues in the Pv domain of Vtgs, excluding Vtg3 and Vtg6, ranges from 44% to 50%, and they are almost all predicted as potential phosphorylation and glycosylation sites. The abundance of highly phosphorylated serine residues is a distinctive feature of the Pv domain [44,45], enabling the Pv domain to effectively bind metal cations such as Ca^2+^, Fe^3+^, Mg^2+^ and Zn^2+^, which is very crucial for embryo survival in many freshwater species because they are unable to obtain metal ions directly from the environment [6,46]. In addition, the presence of multiple glycosylation sites also enhances the transport of carbohydrates with the Pv domain, further enhancing the nutritional function of Vtg [3]. We discovered that the β′-c and Ct domains collectively possess 14 highly conserved cysteine residues, which are known to establish disulfide linkages believed to be essential for the intricate folding of Vtg polypeptides [3,41]. We also found that the β′-c domain contains a CGLC motif, which is involved in interchain disulfide linkages during Vtg dimerization and is necessary for the oocyte’s receptor-mediated endocytosis of the Vtgs [47,48]. In addition, the β′-c and Ct domains have been shown to be homologous to the von Willebrand factor type D domain (vWFD) and have been found to be involved in physiological processes such as immunity and coagulation [49,50]. However, there is a general lack of characterization of the β′-c and Ct domains nutritional functions [9]. The general lack of β′-c and Ct domains in type Ⅰ and type Ⅲ Vtgs may imply that the vWFD domain is not essential for the activity of Sichuan bream Vtgs, and it may function only in the Vtg2 monomer.

### 3.2. The Expression of vtgs Is Adapted to Vitellogenesis in Sichuan Bream

The qRT-PCR results demonstrated that the expression of *vtgs* in Sichuan bream exhibited tissue-specific patterns. The liver was the leading site of *vtgs’* expression, and the *vtg* transcripts were also detected in the gut, ovary, spleen and heart, which was similar to the expression profiles in zebrafish [9], *Gobiocypris rarus* [51] and *Kryptolebias marmoratus* [52]. In oviparous animals, the heterologous synthesis of yolk substances outside the oocytes is commonly observed, as the liver of teleosts [3], the fat body of insects [34] and the intestine of nematodes [35] are the most major expression sites of *vtgs*. Heterologous synthesis reduces the burden on the oocyte because Vtg carries lipids, metal ions and carbohydrates to enter the oocyte to supply energy for offspring, thus avoiding the internal depletion of the oocyte [36,53]. Moreover, the entry of heterologous substances into the oocyte enhances organismal immunity and further favors reproductive health [53]. However, it is noteworthy that the expression profile of *vtgs* in the ovary resembles that observed in the liver during vitellogenesis despite significantly lower levels of *vtg* expression in the ovary. Except for the smaller *vtg3* and *vtg6*, the expression of *vtgs* in the ovary was low prior to vitellogenesis, increased sharply during vitellogenesis and reached a peak during the late vitellogenesis stage, suggesting that Sichuan bream vitellogenin may also originate in the ovary. Studies on *Tanichthys albonubes* [44], *Larimichthys crocea* [53] and *Rhodeus uyekii* [54] support our results that the ovary is also involved in the synthesis of Vtg through the autosynthesis pathway as an important supplement during the period of high demand for yolk production. Vtg produced in the ovary is more efficiently transported to the oocyte than Vtg produced in the liver, as this omits the transport process of Vtg in the bloodstream [55].

We noticed that type Ⅰ *vtgs* exhibited the highest expression levels among all tissues that apparently expressed *vtgs*, particularly *vtg1* and *vtg4*. Type Ⅰ Vtgs (VtgAo1) have been the predominant vitellogenin in studies of other Cyprinidae fish, such as *Tanichthys albonubes* [44], zebrafish [9] and common carp [23]. This class of Vtgs containing only LvH, Pv and LvL domains appears to represent the basic function and characteristics of yolk proteins in Cyprinidae species. The single knockout of any *vtg* in zebrafish results in a compensatory upregulation of a type Ⅰ *vtg* gene (*vtg7*) [56]. We propose that type Ⅰ Vtg maintains the overall functional availability of yolk proteins, although the regulatory mechanisms may be intricate.

### 3.3. Regulation of Vitellogenesis by 17β-Estradiol and Its Receptors

The final step of E_2_ synthesis in vivo requires the conversion of substrate testosterone or estrone catalyzed by aromatase Cyp19a1 [57]. Letrozole inhibits the expression of *cyp19a1* and thus prevents the formation of E_2_ [58]. Our study found that E_2_ is indispensable for the initiation of vitellogenesis in the Sichuan bream, as treatment with letrozole will significantly inhibit the expression of all *vtgs*. Similar to studies in other fish, the inhibition of estrogen formation in vivo further inhibited vitellogenesis [59,60]. Among the groups administered different concentrations of E_2_, the inclusion of 100 μg/g of E_2_ in the diet exhibited the most potent stimulatory effect on vitellogenesis, resulting in the highest levels of *vtg* transcriptional induction, the greatest value of GSI and the most pronounced changes in histological characteristics. Our recent work found that under controlled laboratory conditions, the developmental period required for juvenile Sichuan bream to reach the vitellogenesis stage is 150 days [33]. However, after three weeks of feeding them diets containing 100 μg/g E_2_, the 120-day-old juveniles had already entered the vitellogenesis stage, significantly reducing developmental time.

It is generally accepted that there is a significant dose effect of E_2_ on the induction of Vtg synthesis [44,61]. Interestingly, treatment with higher concentrations of E_2_ (200 μg/g) did not result in greater induction. We reviewed another similar study [62] in which exposure to high concentrations of E_2_ (10μL/L) for 60 days in *Larimichthys polyactis* resulted in delayed ovary and follicular development. Treatment with higher concentrations of E_2_ appears to lead to a disruption of the endocrine system through a negative feedback regulation, which may impede Vtg synthesis and, to some extent, explain our experimental results. Furthermore, upon treatment with a low concentration of E_2_ (10 μg/g), only type Ⅰ *vtg1*, *vtg4* and *vtg5* exhibited significant upregulation, which might be related to differences in the regulation of the *vtg* gene promoters. During vitellogenesis, estrogen forms dimers with estrogen receptors and subsequently binds to the estrogen response elements (EREs) in the promoter region of the *vtg* genes, leading to the initiation of transcription and translation [22]. The number of estrogen response elements (EREs) within the *vtg* promoter region may modulate the transcriptional response to estrogenic compounds. We identified multiple imperfect EREs (ERE-half) in the Sichuan bream *vtg* promoter sequence, and the ERE-half had been confirmed as functional EREs [63,64]. Unlike only one in type Ⅱ and Ⅲ *vtgs*, we found multiple ERE-half sites in the promoter region of all type I *vtgs*, which may make type I *vtgs* more sensitive to low concentrations of E_2_. At the same time, we have also detected several possible transcription factor response elements, including PPAR, GATA, RXR and HNF-3, which have been demonstrated to regulate *vtg* transcription by responding to signals other than estrogen [54,65,66]. Therefore, the regulation of *vtg* expression in vivo represents a highly intricate process that a combination of transcription factors can potentially influence.

The physiological effects of estrogen require binding to estrogen receptors to be exerted [22]. While G protein-coupled estrogen receptor (GPER) has been identified as a modulator of *vtg* transcription in fish [67], the prevailing evidence suggests that nuclear receptors (nERs) primarily mediate the estrogenic effect [68,69]. We supported the induction of vitellogenesis by nERs via drug treatment experiments, which significantly reduced the expression of all *vtgs* after adding the estrogen receptor antagonist ICI to the diet. The estrogen receptor subtypes ERα and Erβ are expressed in the liver of most fish, but they may play different roles in vitellogenesis. It has been reported that ERβ has a higher binding affinity for E_2_ than ERα [70]. Transcription of *vtg* was found to be primarily mediated by ERβ rather than ERα in the study using selective estrogen receptor modulator (SERM) treatment of rainbow trout primary cultured hepatocytes [32]. The research conducted on zebrafish embryos demonstrated that the specific knockdown of *erβs* using morpholinos effectively suppressed the estradiol-induced expression of *vtg* and *erα* mRNA [71]. However, more studies have suggested that ERα predominantly mediates estrogenic effects in fish Vtg synthesis [29,30,72]. ERα expression in the liver increases during the breeding season, which parallels the trend of E_2_ and Vtg levels in the blood [73,74]. Our study supports the role of ERα as the primary regulator of vitellogenesis, as treatment with PPT dramatically enhanced the expression of all *vtgs*. Although DPN treatment upregulated the expression levels of *vtg1* and *vtg4* by 1.6 and 1.9 times, we speculate that this may be due to natural physiological changes rather than the effect of DPN because the expression of *vtg1* and *vtg4* changes during vitellogenesis or under other drug treatment are astonishing. Therefore, the roles of nER subtypes in mediating estrogen action vary among fish species, indicating potential evolutionary differences in the mechanisms regulating Vtg synthesis, which should be the focus of our future research.

## 4. Materials and Methods

### 4.1. Ethics Statement

The experimental protocols were approved by Southwest University, and the study was conducted following the guidelines set forth by the Institutional Animal Care and Use Committee of Southwest University.

### 4.2. Experimental Animals and Drug Treatment

The experimental fish were artificially bred of *Sinibrama taeniatus*, with parents taken from the natural rivers of the Upper Yangtze River. Fish were maintained in indoor tanks equipped with a water circulation system, maintaining a constant temperature of 26 ℃ and a 14L:10D lighting cycle. They were fed twice daily with commercial feed. For gene expression analysis, ovary, spleen, heart, liver (hepatopancreas), skin, gut, brain, gill, kidney and muscle tissue from three adult female fish aged 1 year (body length: 68.43 ± 3.10 mm; body weight: 4.39 ± 0.42 g) and testis tissues from three adult male fish aged 1 year (body length: 71.22 ± 4.03 mm; body weight: 3.92 ± 0.23 g) were collected. In addition, we collected ovarian and liver tissues from juvenile females at 115 (body length: 46.71 ± 2.13 mm; body weight: 1.82 ± 0.17 g), 165 (body length: 52.85 ± 3.32 mm; body weight: 2.36 ± 0.19 g) and 185 (body length: 56.77 ± 1.67 mm; body weight: 2.72 ± 0.12 g) days of development to investigate the expression profile of *vtg* genes during ovarian development. According to our recent research [33], their ovarian developmental stages were categorized as pre-vitellogenesis, vitellogenesis and late vitellogenesis, respectively. The experimental fish were anesthetized with tricaine methanesulfonate (MS-222) prior to sampling, and all excised tissues were subsequently flash-frozen in liquid nitrogen and stored at −80℃.

The following chemicals were used in this study: 17β-estradiol (E_2_, CAS No.: 50-28-2), Letrozole (LTZ, CAS No.: 112809-51-5); an antagonist of E_2_, Fluvestrant (ICI, CAS No.: 129453-61-8); an antagonist of nuclear estrogen receptor (nER), 1,3,5-Tris(4-hydroxyphenyl)-4-propyl-1H-pyrazole (PPT, CAS No.: 263717-53-9); an agonist of the estrogen receptor Erα, 2,3-Bis(4-hydroxyphenyl)propionitrile (DPN, CAS No.: 1428-67-7) and an agonist of the estrogen receptor Erβ. All chemicals were purchased from Sigma-Aldrich (USA) and dissolved in dimethyl sulfoxide (DMSO) as stocks.

Female juveniles (body length: 42.52 ± 2.94 mm; body weight: 1.22 ± 0.26 g) up to 100 days of development were selected for drug treatment. According to our recent finding [33], ovarian development at this time is in the pre-vitellogenesis stage. As described in other studies [75], the above drugs were added to commercial feeds using the ethanol evaporation method. The E_2_ treatment was divided into four concentration groups: 10 μg/g feed, 50 μg/g feed, 100 μg/g feed and 200 μg/g feed. The remaining chemicals were added at a concentration of 100 μg/g feed. The ethanol blended in the control group did not contain any drug. There were 3 replicates per treatment group, and each group contained 20 fish. The treated diets were fed twice daily at a feed weight of 3% of the fish’s body weight. The daily drug intake of the experimental fish was approximately 0.37 ± 0.08 μg/g body weight, 1.82 ± 0.39 μg/g body weight, 3.65 ± 0.78 μg/g body weight and 7.30 ± 1.54 μg/g body weight, respectively, in each treatment group of E_2_ and 3.65 ± 0.78 μg/g body weight per day for the remaining drug treatment groups. After 3 weeks of continuous feeding, the fish were fasted for 24 h before being sampled and dissected under MS-222 anesthesia. The ovaries were removed and weighed to determine the gonadosomatic index (GSI, GSI = [gonad weight/body weight] × 100%) and then fixed in Bouin’s solution for further histological analysis. In addition, the liver was removed and frozen in liquid nitrogen and then stored at -80℃ for further gene expression analysis.

### 4.3. Histology Analysis

The fixed ovarian tissues were dehydrated with serial dilutions of ethanol, embedded in paraffin wax, sectioned serially at 5–8 μm in thickness (Leica RM 2235) and subsequently double stained with hematoxylin and eosin for histological analysis. All micrographs were captured with an optical microscope (Nikon Eclipse 80i). The classification of ovarian developmental stages in Sichuan bream is based on the findings from our recent study [33].

### 4.4. Gene Expression Analysis by Quantitative Real-Time PCR

In this study, the expression profile of the *vtg* gene in Sichuan bream was investigated with quantitative real-time PCR (qRT-PCR), and the primers of the related genes are shown in Supplementary Table S2. Total RNA from each tissue was extracted using an Animal Total RNA Isolation Kit (Foregene, Chengdu, China), and cDNA was synthesized from 1 μg total RNA using SuperScript III reverse transcriptase (Invitrogen, Carlsbad, CA, USA) according to the manufacturer’s protocol. Quantitative real-time PCR (qRT–PCR) was conducted with TB Green ^®^ Premix Ex TaqTM II (TaKaRa Bio, Shiga, Japan) on a QuantStudio 1 (Thermo Fisher, Waltham, MA, USA), following the specific protocols described in our previous studies [76]. The internal reference gene β-actin was utilized, and the expression level of genes was analyzed using the 2^−ΔΔCT^ method. The relative expression of *vtg6* in muscle was used as a control value to assess the relative expression of Sichuan bream *vtg* gene. All of the results included biological replications from three individuals.

### 4.5. In Silico Analyses

In this study, Vtg sequence information was derived from our *Sinibrama Sinibrama* genomic data (unpublished), and the CDS sequence was identified using the online BLAST program on the website of the National Center for Biotechnology Information (https://www.ncbi.nlm.nih.gov/, accessed on 8 October 2023). The schematic diagram of gene structure was drawn by the online software GSDS 2.0 (http://gsds.gao-lab.org/, accessed on 10 October 2023). Transcription factor binding sites in promoter regions were predicted using the online database PROMO (https://alggen.lsi.upc.es/cgi-bin/promo_v3/promo/promoinit.cgi?dirDB=TF_8.3, accessed on 10 October 2023) with the maximum matrix difference rate set to 5. Theoretical molecular weights and isoelectric points were calculated using the Expasy MW/pI tool (https://www.expasy.org/, accessed on 11 October 2023). Signal peptide predictions were made using the online SignalP 4.1 server (https://services.healthtech.dtu.dk/services/SignalP-4.1/, accessed on 11 October 2023). Potential phosphorylation sites, N-glycosylation sites and O-glycosylation sites were predicted using NetPhos 3.1 (https://services.healthtech.dtu.dk/services/NetPhos-3.1/, accessed on 11 October 2023), NetNGlyc 1.0 (https://services.healthtech.dtu.dk/services/NetNGlyc-1.0/, accessed on 12 October 2023) and NetOGlyc 4.0 Server (https://services.healthtech.dtu.dk/services/NetOGlyc-4.0/, accessed on 12 October 2023), respectively. Multiple sequence alignment was performed with ClustalW (https://www.genome.jp/tools-bin/clustalw, accessed on 14 October 2023), and then, a neighbor-joining phylogenetic tree with 1000 bootstrap replicates was constructed using MEGA 7.0 soft (Informer Technologies, Inc., New York, NY, USA). Sequence information for Vtg peptides from related species is provided in Supplementary Table S3.

### 4.6. Statistical Analysis

All data are presented as the mean value ± standard deviation. One-way ANOVA was employed for group comparisons, followed by Duncan’s multiple-range test. A significance level of *p* < 0.05 was considered statistically significant. All statistical analyses were conducted using SPSS 23 software (SPSS, Chicago, IL, USA).

## 5. Conclusions

In this study, six Sichuan bream vitellogenin genes were identified and classified into three types based on their distinct domains: type I (*vtg1*, *4*, *5*, *6*), type II (*vtg2*) and type III (*vtg3*). The liver is the primary tissue in which the *vtg* genes are expressed, with type I *vtgs*, particularly *vtg1* and *vtg4*, being the predominant types. The expression of *vtg* mRNA in the liver and ovary was low prior to vitellogenesis, increased rapidly during vitellogenesis and peaked during the late vitellogenesis stage. Drug treatment experiments demonstrated that *vtg* gene expression in the liver was highly regulated by E_2_. The stimulation of *vtg* expression and ovarian development was most pronounced after three weeks of feeding with E_2_ at a concentration of 100 μg/g, leading to an earlier initiation of the vitellogenesis stage in the ovary. Additionally, in the liver of Sichuan bream, the initiation of *vtg* translation is primarily mediated by E_2_ binding to ERα rather than ERβ. This study provides a comprehensive characterization of the vitellogenin gene family in Sichuan bream, offering a theoretical foundation for regulating ovarian development in cultured fish.

## Figures and Tables

**Table 1:** Detailed information of the identified Vtgs.

Gene Name	Chromosome Site (bp)	Intron Numbers	CDS Length (bp)	Deduced Polypeptide Characteristics		
				Length (aa)	Pi	Mw (kDa)
Vtg1	10,774,548–10,781,219	27	4029	1342	8.64	145.97
Vtg2	10,759,157–10,769,772	33	4848	1615	7.97	177.07
Vtg3	9,957,004–9,971,631	29	3723	1240	7.00	138.94
Vtg4	10,791,557–10,798,302	27	4020	1339	8.57	145.87
Vtg5	10,804,185–10,811,251	27	4023	1340	8.57	145.93
Vtg6	10,748,523–10,755,421	20	3342	1113	9.12	121.99

## Data Availability

Data are contained within the article and Supplementary Materials.

## Supplementary Materials

The following supporting information can be downloaded at https://www.mdpi.com/article/10.3390/ijms25126739/s1.
