# Supplementary material for: Genome-Wide Identification, Expression and Response to Estrogen of Vitellogenin Gene Family in Sichuan Bream (Sinibrama taeniatus)
Source: Int J Mol Sci. 2024 Jun 19;25(12):6739. doi: 10.3390/ijms25126739 (PMC11203743; doi:10.3390/ijms25126739)
Supplement: Supplementary file 1 [file ijms-25-06739-s001.zip › ijms-2998237-supplementary.pdf]

Supplementary File: Genome-wide identification, expression and response to estrogen of vitellogenin gene family in Sichuan bream (*Sinibrama taeniatus*)

Zhe Zhao [1, 2], Li Peng [1, 2], Qiang Zhao [1, 2] and Zhijian Wang [1, 2] *

[1]Integrative Science Center of Germplasm Creation in Western China (CHONGQING) Science City & Southwest University, Chongqing 401329, China

[2]Key Laboratory of Freshwater Fish Reproduction and Development (Ministry of Education), Key Laboratory of Aquatic Science of Chongqing, School of Life Sciences, Southwest University, Chongqing 400715, China

*Corresponding author:

Zhijian Wang, Email address: wangzj1969@126.com

A

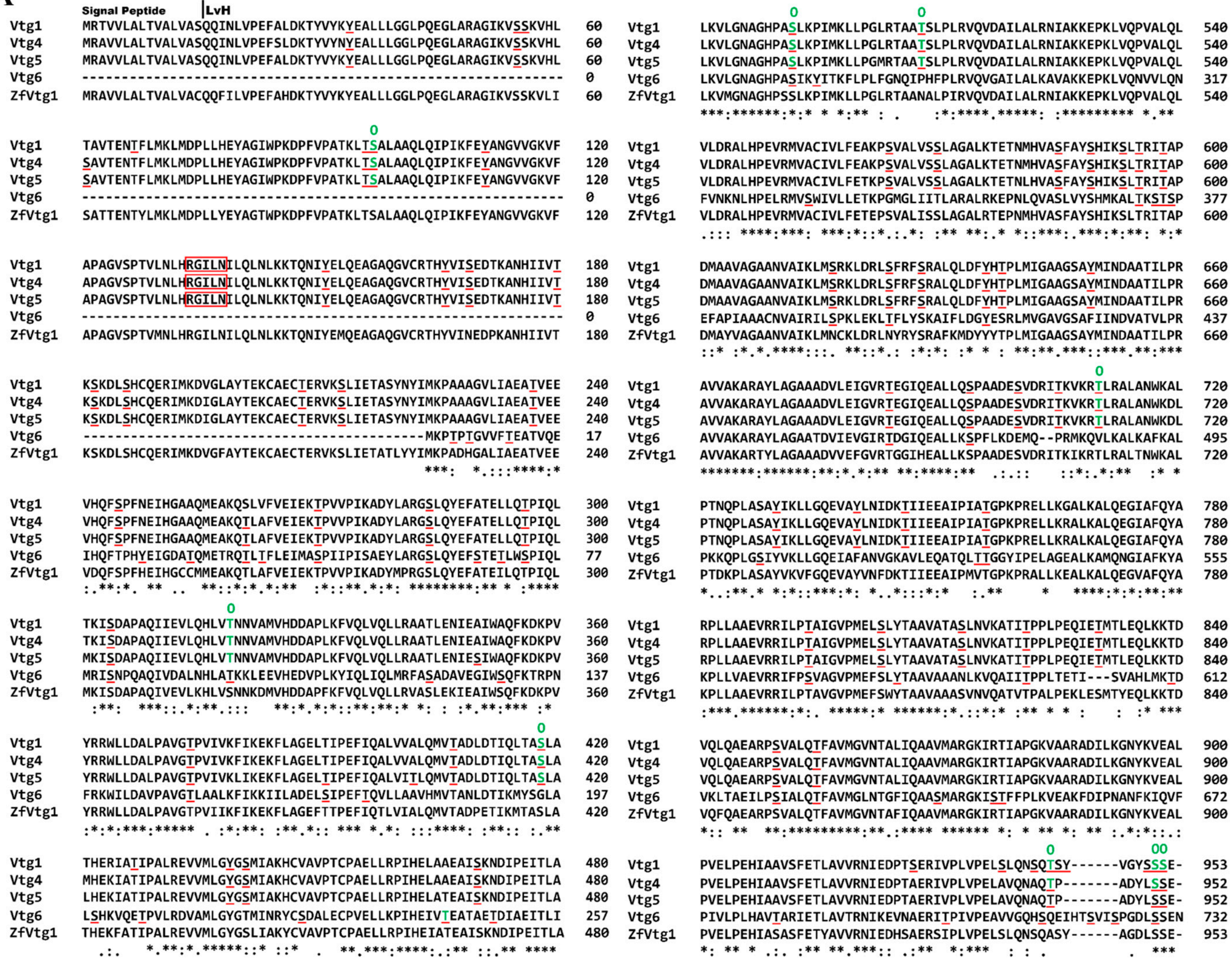

```
                        O    O
Vtg1      ---------IPDETPVRATAPFHKTLCLAVPYIEIKGCIEVHSHNAAFIRNAPLFYIIGQ  1004   Vtg1    --    1342
Vtg4      ---------IPDETPVRATAPFHKTLCLAVPYIEIKGCIEVHSHNAAFIRNAPLFYIIGQ  1003   Vtg4    --    1339
Vtg5      ---------IPDETPVRATAPFHKTLCLAVPYIEIKGCIEVHSHNAAFIRNSPLFYIIGQ  1003   Vtg5    --    1340
Vtg6      VNHLASVMPPAEEPELRTSVPVHRTLCSVVPHIGVKACLEIMSCNADFIKNTTLYNMIGQ  792    Vtg6    SG    1113
ZfVtg1    ---------MSSVASVRAPAPFDRTLCYAVPYIEIKGCVEVHSHNAAFIRNSTLFYIIGH  1004   ZfVtg1  --    1362
                     .   :*: .*..:*** .**:* :*.*:*: * ** **:*: *: :**:
                                                O               |Pv
Vtg1      HSARAAVSRAEGPAVEKLELEVQVGPRAAERLLKQISLIDEETPEGKAFLLKLREILETE  1064
Vtg4      HSARAAVSRAEGPAVERLELEVQVGPKAAERLLKQINLIDEETPEGKAFLLKLREILETE  1063
Vtg5      HSARAAVSRAEGPAVERLELEVQVGPRAAERLLKQINLIDEETPEGKAFLLKLREILETE  1063
Vtg6      HGVHINIARADGPAVEGLELEVQLGPRAAKKLLKEINIVDAKTPKFKTVLQKLKEILEGG  852
ZfVtg1    HSVRAAVARAEGPAVERLELEFEVQVGPRAAERLVKQINIIDDDTPEGQAFLLKLREILDTE  1064
          *..:  ::**:*****  **:***:**:**::*:*:*.::* .**: ::.* **:***:
                OO  OOO OOO   OO O                   OOOOOO OOO|LvL O O
Vtg1      DKNVPVSSESSSS-SSSRI--------------------ISSSSSSMSSSRMTETATIME  1103
Vtg4      DKNVPVSSESS---SSSRI--------------------ISSSSSSMSSSRMTETATIME  1100
Vtg5      DKNVPVSSESSS--SSSRI--------------------ISSSSSSMSSSRVTETATIME  1101
Vtg6      QRNNNSRFFNHNVLFYPRSTSSRS---------------ISSSSSVRS-----------  885
ZfVtg1    AKNAPVSSESSSS-RNSRSSSSRSTSTSASSSLRSSSSSSQSSSSSMSSSRMSKTATIIE  1123
           :*      .      *                      *****: *
                    O    O   OO O   O
Vtg1      PFRKFHKDRYMAPHGASKAKSSGSAASSFEALRKQAKFLGNGIPPVFAVIARAVRVDHKL  1163
Vtg4      PFRKFHKDRYMAPHGASKAKSSGSAASSFEALRKQAKFLGNGIPPVFAVIARAVRVDHKL  1160
Vtg5      PFKKFHKDRYLAPHGASKAKSSGSAASSFEAIQKQAKFLGNALPPVFAVIARAVRVDHKL  1161
Vtg6      -----------SNSHLSKELSSGQNSEHFKPVKEQAIFLGDSLPPVFAIIARVVRVDRKL  934
ZfVtg1    PFRKFHKDRYLAHHSATKDTSSGSAAASFEQMQKQNRFLGNDIPPVFAIIARAVRADQKL  1183
                     :    :*  ***. :  *: :::*  ***: :*****:***.**.*:**

Vtg1      LGYQLAAYFDKPTARVQLVISSIAENDNLKICADGALLSKHKVTAKVAWGPECQQYAVTA  1223
Vtg4      LGYQLAAYFDKPTARVQLVISSIAENDNLKICADGALLSKHKVTARLAWGPECQQYAVTA  1220
Vtg5      LGYQLAAYFDKPTARVQLVISSIAENDNLKICADGALLSKHKVTAKVAWGPECQQYAVTA  1221
Vtg6      LGYQLVAYLDRPTSRVQIIVVSIAVKDNWKMCANGILLSKHEVAARIAWGAERQQYAISI  994
ZfVtg1    LGYQLAAYFDKPTARVQLIVSSIAENDNMKICADGALLSKHKVTGKFSWGAECKQYAVFA  1243
          *****.**:*:**:***::: *** :** *:**:* *****:*:.:.:** * :***:

Vtg1      KAEAGVLGEFPAARLELEWERLPLIVTTYAKKLSKHIPMAALQAGFRLERATNSEKEIEL  1283
Vtg4      KAEAGVLGEFPAARLELEWERLPLIVTTYAKKLSKHIPMAALQAGFRLERATNSEKEIEL  1280
Vtg5      KAEAGVLGEFPAARLELEWERLPLIVTTYAKKLSKHIPMAALQAGFRLERATNSEKEIEL  1281
Vtg6      SAGAGNHGPSPMAYLKVKCEKVPTIITTYAKSLAEYIPGVAQMTGLAISDEKNIERQIEL  1054
ZfVtg1    KAEAGVLGEFPAARLEVEWERLPIIVTTYAKKLGKHILTAAYDTGFRFERATNSEKEIEL  1303
          .* **  *  * * *::: *::* *:*****.*.::*  .*  :*: :.  .* *::***

Vtg1      TAALPTQRSLNVIARIPEMTLSRMAIPLPVAVPINPDGTLSIHIDEDILSWIQKHINEE-  1342
Vtg4      TAALPTQRSLNVIARIPEMTLSRMAIPLPVTVPINPDGTLTIHIDEDILSWIQKHIKEE-  1339
Vtg5      TAALPTQRSLNVIARIPEMTLSRMAIPLPVAVPINPDGTLTIHIDEDILSWIQKHIKEE-  1340
Vtg6      VIVVPTERTLDIIIKTPTMTLSKSNLVLPIALTFEAVPAYPE---EDVMNIIRNLYVEAS  1111
ZfVtg1    TAALPSQRSLNIIARIPEITMSKRDIYLPVAVPINPDGTFSIETYEDFLAWIQKYIKEE-  1362
          . .:*::*:*::* : * :*:*:  : **::: ::    :      **.:  *::    *
```

B

```
        Signal Peptide  |LvH
Vtg2    MRAVVLALTLAFVASQQINIVPEFALDNTYVVYKYEALLLGGLPQEGLARAGIKVSSKVHL   60
ZfVtg2  MRAVVLALTLALVASQQNKLVPEFAHDKTYVVYKYEALLLSGIVQEGLAKAGIQIKSKVLL   60
        ***********:*****  ::***** *:***********.*: *****:***::.*** *

Vtg2    SAVTENTFLMKLMDPLLHEYAGIWPKDPFVPATKLTSALAAQLQIPIKFEYANGVVGKVF   120
ZfVtg2  SAATENTFLLKFVDPLFYEYAGTWPKDQVFPATKLNSALAAQLQTPIKFEYANGVVGKVF   120
        **.******:*::***::**** ****  ..*****.******** ***************

Vtg2    APAGVSTTVLNLHRGILNILQLNLKKTQNIYELQEAGAQGVCKTQYVISEDTKANHIIVT   180
ZfVtg2  APAGVSPSVLNLYRGILNILQLNFKKTQNIYELQEAGAQGVCKTQYVISEDPKADRITVT   180
        ******  :****:**********:*************************** **::* **

Vtg2    KAKDLSHCQERIMKDVGLAYADRCAECTKRVKSLTETASYNYIMKPSAAGVLIAEATVEE   240
ZfVtg2  KSRDLSHCQERIVKDIGLAYTETCVECTKRIKSLIETANYNYIIKPSDSGALIAEATVKE   240
        *::*********:**:****::  *.*****:***  ***.****:***  :*.*******:*

Vtg2    VHQFSPFNEIHGAAQMEAKQTLAFEEIQKTPVVPIKADYLARGSLQYEFATEILQTPIQL   300
ZfVtg2  VHQFSPFNEIHGAAMMEATQSLVFVEFEKAPVVPVKADYMPRGSLQYEFATEILQTPIQL   300
        ************** ***.*:*.* *::*:****:****: *******************

Vtg2    MKISDAPAQIVEVLQHLVVNNVAMVHDDAPLKFVQLVQLLRAATLENIEAIWAQFKDKPV   360
ZfVtg2  TKISNAPAQIKEVLKHLVENNVAMVHDDAPLKFVQLVQFLRAATLKDTEAIWAQFKDKPV   360
         ***:***** ***:*** *******************:******:: ************

Vtg2    YRRWLLDALPAVGTPVIVKLIKEKFLAGELTIPDFIQALVVALQMVTADLDTIQLTASLA   420
ZfVtg2  YRRWLLDTLPAVATPVVLKFIKEKFLAGEFTLTEFIPTLVVALQMVPADLETIQWTASLA   420
        *******:****.***::*:*********:*:  :** :******** ***:*** *****

Vtg2    LHEKIATIPALREVVMLGYGSMIAKHCVAVPTCPAELLRPIHELAAEAISKNDIPEITLA   480
ZfVtg2  LHEKIATIPALREVVMLGYGSMIAKYCVAVPTCPAELLRPIHEIATEAISKNDIPEITLA   480
        *************************:*****************:*:**************

                    O
Vtg2    LKVLGNAGHPASLKPIMKLLPVLKTAAASVPIRVQVDAILALRNIAKKEPKLVQPVALQL   540
ZfVtg2  LKVLGNAGHPSSLKPIMKLLPVLKTTASALPIRVQVDAILALRSIAKKEPKLVQPVALQL   540
        **********:*************:*:::*************.****************

Vtg2    VLDRALHPEVRMVACIVLFEAKPSVALVSSLSGALKMETNMQVASFAYSHIKSLTRITAP   600
ZfVtg2  VLERALHPELRMVACILLFETKPSVAVMSSLAGALRMETNMQVASFAYSHIKSLTRITAP   600
        **:******:******:***:*****::***:***:************************

Vtg2    DMAAVAGAANVAIKLMSRKLDRLSFRFSRALQLDFYHTPLMIGAAGSAYMINDAATILPR   660
ZfVtg2  DMSSVAGAANVAIKLMNRKLDRLNFRYSRAFQLDFYHTPLMIGAAGSAYMINDAATILPR   660
        **::************.******.**:***:*****************************

                                                         O
Vtg2    AVVAKARAYLAGAAADVLEIGVRTEGIQEALLKSPAADESVDRITKIKRTLRALINWKAL   720
ZfVtg2  AVIAKARAYLAGAATDVIEFGLRTEGIQEALLKSPAADESVDRITKIKRTLRALTNWKAM   720
        **:***********:**:*:*:********************************** ****:

Vtg2    PTNQPLASAYVKLLGQEVAFARIDRNIIEQAIPLATGPKPRELLKGALKALQEGIAFQYA   780
ZfVtg2  PSNKPLASAYVKVFGQEMAFGRIDKTIIEQALPMVNGPKPRALLKEALKALQEGISWQRA   780
        *:*:********::***:**.***:.*****:*:..***** *** *********::* *

Vtg2    KPLLVAEVRRIFPTAVGVPMELSMYSAVVGAASVNVKATITPPLPEEIETMTLEQLKKTD   840
ZfVtg2  KPLLVAEVRRILPTSVGLPMELSMYSSVVGAATVNVQATITPPLPEQIETMTLEQLKKTD   840
        ***********:**:**:********:*****:***:*********:*************

Vtg2    VQLQAEARPSIAIQKFAVMGVNTALIQAAVMAKGEIRLIAPGKVAARADILKGNYKVEAL   900
ZfVtg2  VQFKAEARPSIAIHKFAVMGVNTAFIQAAVMARGKIRTIAPGKVAARADILKGNYKIEAL   900
        **::*********:**********:*******:*:** ******************:***

                                             O O
Vtg2    PVELPEHIAAVSFETLAVVRNIEDPTAERIVPLVPELSLQNPQ--TWSSEIPDPDMRAEA   958
ZfVtg2  PVELPEHIASASFETFAFARDVEDPSAERTVPLVSVLSLQNRQSQRLSSENADSEIETET   960
        *********:.****:*..*::***:*** ****   ***** *    ***  * ::.:*:

Vtg2    PMRPTAPFHKTLCLAVPYIEIKGCIEVHSHNAAYIRNSPFFYIIGQHSARATVARAEGPT   1018
ZfVtg2  PVKAPAPFDRTLCYAVPYIEIKGCVEVHSHNAAFIRNSTLFYIIGQHSARAAVARAEGPA   1020
        *::   ***.:*** **********:********:****  :***********:*******:

                                   |Pv            OO  OOOO
Vtg2    VERLELEVHVGPRAAERLLKQISFIDEETPEGKAFLLKLREILETEDKNVPVSSESSSSS   1078
ZfVtg2  VEKLEFEVQVGPRAAERLAKQINIIDDETPEGKDFLLKLREILETESKNATVSSGSSSSR   1080
        **:**:**:********* ***.:**:****** ************.**. *** ****

           OOOO                 OO OOO  O  |LvL O
Vtg2    RIISSSS----------------SSMSSSRVTELMGFQTANHMEPFKKFHKDQYMAEHGA   1122
ZfVtg2  SSSSSRSSSSSSSTSNSTSSSSSSSSSSSMSSSRWSKTLTKMEAFRKFHKDQYKTHHGD   1140
           ** *                   ** *** ::.    :* .:** *:*******  :.**

        O   O
Vtg2    SKAVSSGSAASSFEHIQKQAKYLGNAVPPVVAVIARVVRVDHKLLGYQLAAFFDKPTARV   1182
ZfVtg2  SKS--SRSTGSSLEQIQKQSRYLGNTVPPVFAIIARAVRVDRKLLGYQFVAFFDKPSSRV   1198
        **:   * *:.**:*:****::****:****.*:***.****:******:.******::**

                                 OO
Vtg2    QLIVSSIAENDNWKLCADGVLLSKHKVTTKVAWGAECQQYAVITKAEAGILGEFPAVRLE   1242
ZfVtg2  QFIASSIAENDNFKFCADGVLLSKHKVTSKVTWGAECKEYAVTTKAEAGLLGEFPAFRLE   1258
        *:*.********:*:*************:**:*****::***  ******:******.***

                  O
Vtg2    WEYERLPVIVTTYAKKLSKHIPMAALQAGMRFERATNSEKEIELTVALPTKRTLNVVVRA   1302
ZfVtg2  WEWERLPIIFTTYAKKLSKHIPMAALQAGFNVERAKNSEKELELTVALPSKRTLNVIVRV   1318
        **:****:*.******************:..***.*****:*******:******:**.

                                             |β'-c
Vtg2    PEMTLSRMAIPLPITVPINPDGTLSIHIDQDILFRAQNYIYDYTTAQCSMMQDTITTFNN   1362
ZfVtg2  PEMTMSRMDIPLPVTFPINPDGTFDVHFYEDIYFRAQNYIYDYTTAQCSMMQDTISTFNN   1378
        ****:*** ****:*.*******:.:*: :** **********************:****

Vtg2    RRYKNEMPISCYQVLAQDCTPELKFVVLLKKDEESEENRLNVKLADIDVDLYALGTDAKV   1422
ZfVtg2  KTYKNEMPISCYQVLAQDCTSELKFVALLKKDEESEKTHLNVKLVDIDIDLYTLGTDAKV   1438
        : ***************** *****.*********:.:*****.***:***:*******

Vtg2    KVNEMEVPVSSLPYQHPSGSIQIRKKADGLSLYAPSHGLQEVYFANGHWKIQVADWMKGQ   1482
ZfVtg2  KINGLEVPISSLPYQHPSGSIQIREKADGLSLYAPSLGLHEVYFANGDWKIQVADWMKGQ   1498
        *:* :***:**************:********** **:*******.************

                  |Ct OO        O      O                 O
Vtg2    TCGLCGKADGEIGQEYTTPSGYLTKSSVSFAHSWVLPVESCRDASQCRMKLESVKLEKQA   1542
ZfVtg2  TCGLCGKADGEIKQEYTTPSGYLTESSVSFAHSWVLPAESCRDASQCRMKLESVKFEKQV   1558
        *********** ***********:************.*****************:***.

                  O                                          OO
Vtg2    ILNGQESKCYSVEPVLRCLPGCAPIRTIPVTIGYHCLSTDSNLNMFDGIYEKSVDLRETT   1602
ZfVtg2  ILNGQESKCYSVEPVLRCLPGCAPVRTTPVTVGYHCMSTASNLNMLAGIYEKSVDLRETT   1618
        ***********************:** ***:****:** *****: *************

Vtg2    DAHVACRCTEQCA   1615
ZfVtg2  DAHVACRCTEQCA   1631
        *************
```

C

```
                     |LvH
Vtg3    -------------MANILEPFLNSKKTYEYKYEGLVRVGRELPDLVESALKLRCTFKIIG   47
ZfVtg3  RLCLCLLVALAASEMANYEPFLNSQKTYEYKYEGLVQVGRELPHLVESALKLRCTFKIIG   60
                     ******:***********:******.*****************

Vtg3    ESPQTFVLQISNVDFEDFNGIPGKGVFSPSQKLTKRLSAEFSQPIVFEFSKGQISDIRTA   107
ZfVtg3  ESPHTFVLQVSNVDFEDFNGIPGKSVFSPSKNITKYLSAEISQPIISEYSKGQITDIRTA   120
        ***:*****:**************.*****:::** ****:****: *:*****:*****

Vtg3    PGVSNSVVNIVRGILGFLQVTVKTTQSFYELVELGIHGVCQSSYTVEEDSNAKELIVTQM   167
ZfVtg3  PGVSNTVVNIVRGILGFLQVTVKTTQSFYELIELGIHGLCQSSYTVDEDSNPKELIVTRI   180
        *****:*************************:******:*******:**** ******::

Vtg3    VDINNCQQPAALYSGMALAPEDKLSKQVCVKFHYS--------SLQSLGPISQYLGHFFK   219
ZfVtg3  VDITNCQQPASLYRGMALAPEDKLSKQRGESVVSTVKHTYTVKSTADGGQITKAFA----   236
        ***.******:** *************    ..  :        *  . * *:: :.

                                            OO      O           O
Vtg3    TLHAVLQLSSWH--SNLITGTLKWNTVRVILLNPHTTRQRNVKTHKCKNKGNVEIKKYSE   277
ZfVtg3  ------QERQYFSPFNVKGGNFRMLALRDIELLKVSDTTDKVVTGQVQSRGNLMYKTNKD   290
              *   .:.   *:  *.::  ::* * *    :    :* * : :.:**:  *. .:

Vtg3    YISLHMCKLRGENVVSTILDLIKRLAQANIYHVDSASSTDILDLIQLLRVETLENLEQLW   337
ZfVtg3  LKPIPVVMLNLNDPVPKILDLIKRLAQANIYHVDSETSTEILDLIQLMRVTTLDNLEHLW   350
          : :  *. :: * .******************  :**:*******:** **:***:**

Vtg3    KQVSGNDEHRRWFLDLVVEATDERILKFLETRFKVGDITANEAGQALVVAFNHLSAEPVS   397
ZfVtg3  KQVSGNDEHRRWFLDLVVEVTDERILKFLEARYKAGDITANEAGQALVVAFNHLSAEPVS   410
        *******************.**********:*:*.*************************

Vtg3    VALAQEFLTIPFSKSQPLLWNTVVLAYGSLLYRYCVYTDPCPVTVVQPLLDMAASSLSKN   457
ZfVtg3  VALAQESLTIPFSKSHPLLWNTVVLAYGSLVHRYCVYTDPCPITVVQPLLNMAASSLSKN   470
        ****** ********:***************::***********:*******:*********

                                    O
Vtg3    SEEDMVLTLKALGNAAHPSSIKTLLKFLPGYSAGAEKLPTRVQGAAVQAFRLLAGRDPHS   517
ZfVtg3  SEDEMVLALKSLGNAAHLSSIKTLLKFLPGYSNGAEKLSTRVQGAAVQAFRLLASRAPHS   530
        **::***:**:****** *************** ***** ****************.* ***

Vtg3    VQDIVLNLFVQKNLPAEIRMLACMVLLETKPSIALISVITEVLLEEIDLQVASFSYSLLK   577
ZfVtg3  VQDIVLNLFVQKHLPAEIRMLACIVLLETMPSTALISVVSEVLLEEADLQVASFSYSLLK   590
        ************:**********:***** ** *****::****** *************

Vtg3    GIAKSRTPDNQHLSTACNIAMKILTRKLGHLSYRYSKSMHFDWFHDDFLFGTSTDVYILQ   637
ZfVtg3  GFAKSRTPDNQHLSIACNIAMKILTRKLGHLSYRYSKNLHFDWFHDDFLFGTSADVYMLQ   650
        *:************ **********************.:***************:***:**

        N                                                   O
Vtg3    NESLIPSKLMLKGKIHFIGRILQCLELGVHADGIKELFAGKIPQLKKDLGVTDFATILKI   697
ZfVtg3  NESPIPTKLMLKGKFHFIGRILQFLEFGIRADGLKDLFAGKIPELTKDLGISDLASILKI   710
        *** **:*******:******** **:*::***:*:******:*.****::*:*:****

                                                O
Vtg3    LSDWQNLPKDKPLLTAYIRLFGQEAFLMDVSGDFIQSITKSLSPSAGKESKVWEVIQDVQ   757
ZfVtg3  LSNWQSLPKDKPLLTAYARVFGQEAFLMDVSRDSVQSIIKSFSPSAGKESKVWERIQDVQ   770
        **:**.*********** *:********** * :*** **:************ *****

Vtg3    KGISWHWTKPHLVYEARFIQPTCLGLPVEISKYYSIVNAVTMKAKAEINPPPKEHLDELL   817
ZfVtg3  KGTSWHWTKPHLVYEARFIQPTCLGLPVEISKYYSVVNAVTMQAKAEINPPPKEHLGELL   830
        ** ********************************:******:*************.***

Vtg3    RSDISLQTDGFAGVTKDNFVFHGINIDLFQCGVELKSKIVTSLPWAFGLKINIIEQKYEM   877
ZfVtg3  SSDISMQTDGFIGVTKDHFLFHGINTDLFQCGTELKSKVSMGLPWAFDLKINPKEQKYEM   890
         ****:***** *****:*:***** ******.*****:  .*****.****  ******

                                                            OO
Vtg3    NLTPSKTDTELFSVNSNVFTVLRNIEDPSLSKITPMMPETENSQQGLPLARRILPTSRDE   937
ZfVtg3  NLTPSKSVTELFSVSSNVYAVSRNIEDPTSSKITPMMPETGESWQGVPL--RMLPPLRDE   948
        ******: ******.***::* ******: ********** :* **:**  *:**  ***

Vtg3    QTEDNEVKFRQCAEAKIYGTAICIEAEAKRSHYLHEYPLYYFLGHTRFSYQLEPAKSTKP   997
ZfVtg3  QSKKSGMKFRQCAEAKIYGTALCIEAEAKRAHYLHEYPLYYLLGDTHFSYSLEPAKDAKP   1008
        *:::. :**************:********:**********:**.*:***.*****.:**

                         O  O                   NO       |LvL
Vtg3    IEKIQIQVTAGMKHPPGVSEMMDLSRRVFKDTRDEITSCGEHNLSSSFPANQDLDSTPNP   1057
ZfVtg3  IEKIQIQVSASRQHPSVMSGMVNLNQRVFKEKRDENTSCEERKTSSSLPVTQDLDVTPDP   1068
        ********:*. :**  :* *::*.:****:.*** *** *:: ***:*..**** **:*

Vtg3    VVTVKALGLSPPAKPLGYEGVAFYLPIAQRDDIEMIVSEVGEEANWKMCANANVDKSYSS   1117
ZfVtg3  VVTVKALSLSPQAKPLGYEGVAFYLPTAQKDDIEMIVSEVGEEANWKMCANAHFDRTHTS   1128
        *******.*** ************** **:***********************:.*::::*

Vtg3    AKAHLRWGAECQTYDVSMRVSSACQPESKTSIYTKINWGALPSMFTTIGQRIQEYVPGMS   1177
ZfVtg3  AKAHLRWGAECQTYDVSMRVSAACQPESKPSISTKINWGTLPSVFTTVGQIVQEYVPGVS   1188
        *********************:******* ** ******:***:***:** :******:*

                                                               N
Vtg3    YFMGFYQKYEKNPERQAAVTVVASSPETFDMKVKIPERTIYKKAIPSPIELVGFEAVNLT   1237
ZfVtg3  YIMGFYQKNEENPERQASVTVVASSPETFDLKVKIPERTIYKKKIPSPIELVGIEAANLT   1248
        *:****** *:******:************:************ *********:**.***

Vtg3    ASA     1240
ZfVtg3  MST     1251
         *:
```

Supplementary Figure S1. Alignments of the deduced amino acid sequences of Sichuan bream and zebrafish (Zf) (A) type I, (B) type II and (C) type III Vtgs. Numbers on the right indicate amino acid residue positions. The expected primary cleavage sites for yolk protein (YP) are indicated above the row of sequence by vertical lines followed by the abbreviated name of the YP domains (LvH, Pv, LvL, β'-c, or Ct). Asterisks (*) under sequences indicate identical amino acids, semicolons (:) indicate residues with higher similarity, and periods (.) indicate residues with lower similarity. Dashes indicate gaps inserted for optimal alignment. The conserved RGILN/G, CGLC, QEY, and KLR/KEIL motifs are enclosed by red, black, blue, and orange boxes, respectively. The 14 conserved cysteine residues of the β'-c and Ct domains are highlighted in gray. Predicted O-glycosylation and N-glycosylation sites are indicated by the (O) and (N), respectively, shown above the sequences with the symbol for the relevant residues also set in green typefaces. Predicted phosphorylation sites are underlined in red.

Supplementary Table S1. Sequence consistency percentage (%) matrix of Vtgs

	St Vtg1	St Vtg4	St Vtg5	St Vtg6	Carp VtgAo1	Zf Vtg1	Croaker VtgAa	Bass VtgAa	St Vtg2	Carp VtgAo2	Zf Vtg2	Croaker VtgAb	Bass VtgAb	St Vtg3	Carp VtgC	Zf Vtg3	Croaker VtgC	Bass VtgC
StVtg1	100	97.7	97	42.2	89.3	82.8	41.6	44.2	73.2	71.1	65	42.8	43.4	23.4	25.5	24.9	26.2	25.5
StVtg4	97.7	100	98.1	42.4	89.9	82.4	41.5	44.1	72.9	71.1	64.7	43	43.4	23.3	25.5	24.7	26	25.4
StVtg5	97	98.1	100	42.8	89.8	82.8	41.7	44.3	73.2	71.1	64.8	43.2	43.6	23.1	25.5	24.4	26	25.4
StVtg6	42.2	42.4	42.8	100	42.1	41	28.8	30.2	34.5	35	33	30.1	29.6	18.6	19.3	18.7	18.9	17.8
CarpVtgAo1	89.3	89.9	89.8	42.1	100	82	41.8	44.1	70.5	72.6	64.5	43.7	44.1	22.7	25.2	24.3	26.1	25.9
ZfVtg1	82.8	82.4	82.8	41	82	100	40.1	42.7	65.3	65.8	65.7	42	42.3	22.3	24.7	23.9	25.5	25.4
CroakerVtgAa	41.6	41.5	41.7	28.8	41.8	40.1	100	76.4	51	51.8	50.4	56.4	56.5	17.5	18.9	18.7	20.8	20.4
BassVtgAa	44.2	44.1	44.3	30.2	44.1	42.7	76.4	100	53.9	55.2	52.4	58.9	59.2	18.3	19.9	19.3	21.2	20.8
StVtg2	73.2	72.9	73.2	34.5	70.5	65.3	51	53.9	100	87.8	80.5	51.9	52.6	19.6	21.1	21	21.5	21.1
CarpVtgAo2	71.1	71.1	71.1	35	72.6	65.8	51.8	55.2	87.8	100	80.1	54	54.6	19.7	21.3	20.9	21.8	21.4
ZfVtg2	65	64.7	64.8	33	64.5	65.7	50.4	52.4	80.5	80.1	100	50.6	51.4	18.9	20.5	19.8	21.2	20.6
CroakerVtgAb	42.8	43	43.2	30.1	43.7	42	56.4	58.9	51.9	54	50.6	100	81.4	18.8	20.3	19.7	22.2	21.8
BassVtgAb	43.4	43.4	43.6	29.6	44.1	42.3	56.5	59.2	52.6	54.6	51.4	81.4	100	18.8	20.6	20	22.3	21.9
StVtg3	23.4	23.3	23.1	18.6	22.7	22.3	17.5	18.3	19.6	19.7	18.9	18.8	18.8	100	80.1	76.4	45.1	45.6
CrucianVtgC	25.5	25.5	25.5	19.3	25.2	24.7	18.9	19.9	21.1	21.3	20.5	20.3	20.6	80.1	100	82.1	48.3	48.7
ZfVtg3	24.9	24.7	24.4	18.7	24.3	23.9	18.7	19.3	21	20.9	19.8	19.7	20	76.4	82.1	100	46.7	47.8
CroakerVtgC	26.2	26	26	18.9	26.1	25.5	20.8	21.2	21.5	21.8	21.2	22.2	22.3	45.1	48.3	46.7	100	80.4
BassVtgC	25.5	25.4	25.4	17.8	25.9	25.4	20.4	20.8	21.1	21.4	20.6	21.8	21.9	45.6	48.7	47.8	80.4	100

The comparison between types I, II, and III Vtgs are highlighted with yellow, green, and blue shadows, respectively. St: Sichuan bream (*Sinibrama taeniatus*), Carp: Common carp (*Cyprinus carpio*), Zf: Zebrafish (*Danio rerio*), Croaker: Large yellow croaker (*Larimichthys crocea*), Bass: Striped bass (*Morone saxatilis*).

Supplementary Table S2 Primer sequences for qRT-PCR

Gene names	Upstream Primer (5'–3')	Downstream Primer (5'–3')
vtg1	5'CGCTGAAAATGACAACCTGAAG3'	5'TGGCATAGGTGGTGACGATTAG3'
vtg2	5'TGCTGGTCACCCTGCTAGTCT3'	5'TCTTTCTTGGCAATGTTCCTCA3'
vtg3	5'TCGGTGAATCGCCACAAAC3'	5'GCTGAACTCGGCAGATAGACG3'
vtg4	5'TGACAAGCCAACCGCAAGA3'	5'GGCATAGGTGGTGACGATTAGTG3'
vtg5	5'CCGAGTTCATTCAGGCTCTTGT3'	5'GCTGGGATTGTGGCGATTT3'
vtg6	5'CCTAGAAGCACCAGTAGCCGC3'	5'GGAAGTGAGTCCCCAAGGAATAT3'
β-actin	5' CGAGCTGTCTTCCCATCCA3'	5'TCACCAACGTAGCTGTCTTTCTG3'

Supplemental Table S3. Sequence information and accession numbers of vitellogenins for species shown in the neighbor-joining phylogenetic tree.

Species	Vtg type	cDNA full length (bp)	Amino acida residue (aa)	Accession number
Haliotis discus hannai	Vtg	7753	2391	AB360714
Ichthyomyzon unicuspis	VtgABCD	5976	1823	M88749
Cyprinus carpio	VtgAo1	4098	1353	AB331884
	VtgAo2	5000	1624	AB106873
Carassius auratus	VtgAo1	4241	1290	MT409425
	VtgC	4207	1203	MT409426
Danio rerio	Vtg1	4431	1363	NM_001044897
	Vtg2	5002	1631	NM_001044913
	Vtg3	3970	1253	NM_131265
	Vtg4	4232	1358	NM_001045294
	Vtg5	4225	1358	NM_001025189
	Vtg6	4244	1374	NM_001122610
	Vtg7	4212	1358	NM_001102671
	Vtg8	4489	1496	NC_007133
Rhodeus uyekii	VtgAo1	4275	1424	KM111547
Fundulus heteroclitus	VtgAa	5198	1704	U07055
	VtgAb	5166	1687	U70826
Oryzias latipes	VtgAa	5037	1678	AB064320
	VtgAb	5323	1725	AB074891
Sparus aurata	VtgAa	5143	1675	HG794235
	VtgAb	5252	1709	HG794236
Pagrus major	VtgAa	5239	1715	AB181838
	VtgAb	5348	1716	AB181839
	VtgC	4128	1272	AB181840
Morone americana	VtgAa	5752	1667	DQ020120
	VtgAb	5156	1682	DQ020121
	VtgC	4243	1275	DQ020122
Morone saxatilis	VtgAa	5216	1663	HQ846509
	VtgAb	5145	1682	HQ846510
	VtgC	4050	1275	HQ846511
Larimichthys crocea	VtgAa	5278	1717	KM362364
	VtgAb	5202	1695	KM405515
	VtgC	4236	1254	KM362365
Salmo salar	VtgAs	5127	1659	XM_014168660
Oncorhynchus clarkii	VtgAs	5003	1659	JX683452
	VtgC	3899	1281	JX683453
Anguilla japonica	VtgAe1	5395	1742	AY775788
	VtgAe2	5356	1734	AY423445
	VtgAe3	5423	1759	AY423444
Conger myriaster	VtgAe	5110	1663	AB185334
Gadus chalcogrammus	VtgAa	5195	1663	AB568267
	VtgAb	5098	1648	AB568268
	VtgC	4283	1268	AB568269
Melanogrammus aeglefinus	VtgAa	5189	1665	AF284035
	VtgAb	5103	1643	AF284034
Paralichthys olivaceus	VtgAa	5021	1658	XM_020081193

	VtgAb	5060	1663	XM_020081206
	VtgC	4287	1275	XM_020080687
Hippoglossus hippoglossus	VtgAa	4902	1633	EF582606
	VtgAb	4944	1647	EF582607
Cynoglossus semilaevis	VtgC	4410	1275	XM_008329605
Mugil cephalus	VtgAa	5190	1699	AB288932
	VtgAb	5216	1699	AB288933
	VtgC	4037	1272	AB288934

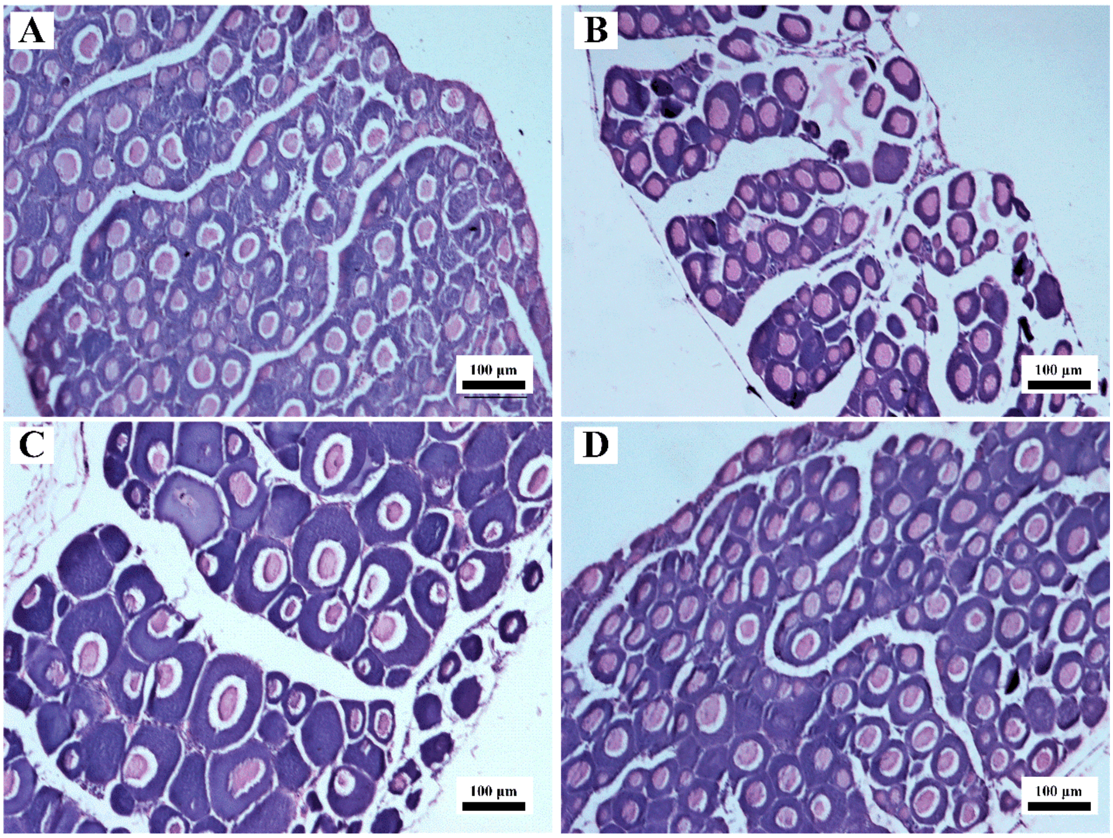

Supplementary Figure S2. Histological pictures of the ovaries of each group of experimental fish after treatment with SERMs. (A) Control group. (B) ICI-treated group. (C) PPT-treated group. (D) DPN-treated group.
